# Proton Pump Inhibitor Usage in Urban vs. Rural Intensive Care Units: A Narrative Review of Implications for Standardization of Care

**DOI:** 10.7759/cureus.71446

**Published:** 2024-10-14

**Authors:** Gaurav Prabhu, Michael Murray, Sarah J Scherbring, Sainamitha R Palnati, Saajan Bhakta

**Affiliations:** 1 Department of Internal Medicine, Thomas Jefferson University Hospital, Cherry Hill, USA; 2 Department of Research, Kansas College of Osteopathic Medicine, Wichita, USA

**Keywords:** gastrointestinal bleeding, intensive care unit, intensive care unit (icu) stay, ppi overutilization, ppi usage in rural population, proton pump inhibitors (ppi), rural vs urban, stress ulcer prophylaxis

## Abstract

Intensive care unit (ICU) patients frequently require and benefit from stress ulcer prophylaxis (SUP) using proton pump inhibitors (PPIs). Despite recognized benefits, PPIs are overutilized in patients who do not have high-risk factors predisposing them to clinically significant gastrointestinal bleeding (CSGIB), including mechanical ventilation and coagulopathy. This overuse increases the risk of adverse effects associated with PPIs. Several urban healthcare systems have created educational initiatives aimed at reducing PPI usage in patients who do not meet recommendations or who are outside the period for serious risk of CSGIB. However, there was no available literature exploring PPI use or educational trends in rural hospitals. This situation presents an opportunity to investigate the disparities in PPI use between rural and urban healthcare settings.

This narrative review aimed to assess current data on PPI usage in both urban and rural critical care environments, and to appraise existing practices, ultimately identifying gaps in current literature and informing future guidelines. With these evaluations, this review intended to provide a comprehensive overview of current PPI prescribing practices in the ICU and improve patient care across diverse healthcare settings.

## Introduction and background

Proton pump inhibitors (PPIs) are widely utilized medications available over the counter, by prescription, and in hospital settings to reduce gastric acid production and treat gastrointestinal disorders. These medications are especially common in the intensive care unit (ICU) due to their effectiveness in preventing stress ulcers, which can lead to clinically significant gastrointestinal bleeding (CSGIB) and higher mortality rates among this in-patient population [1]. Only a small subset of ICU patients with additional complicating factors are at risk for gastrointestinal bleeding [2]. The majority of hospitalized patients are not recommended for stress ulcer prophylaxis (SUP) with PPIs, particularly in non-ICU settings [2,3].

The utilization of PPIs in hospitalized patients as a whole is on the rise globally, despite only a limited number of patients meeting requirements for possible benefit from SUP [1,3-5]. In ICUs worldwide, up to 60% of PPI prescriptions are started with no reasonable indication such as previous gastrointestinal bleeding, known gastroesophageal reflux disease, mechanical ventilation, or coagulopathy [2,6-7]. Many of these prescriptions are continued beyond the necessary therapeutic duration or fail to be discontinued upon discharge [6-7]. This raises additional concerns due to potential side effects, interactions with other medications, and financial implications for the patient [1,3]. New evidence indicates increased risks in and outside the gastrointestinal system associated with long-term PPI use, particularly among older populations and those with comorbidities, including autoimmune diseases, chronic kidney disease, cirrhosis, and cardiovascular disease [3,8-9]. Responsible prescribing and frequent reevaluation are some of the most universal ways of combating excessive use.

Numerous initiatives have been implemented to address the inordinate and ineffective use of PPIs in hospitalized patients, predominantly educational interventions supplemented by various targeted strategies for providers [5]. Limited evidence exists regarding PPI overutilization in rural hospital settings or any differences in recommendations outside large, high-resource ICUs. This narrative literature review aims to assess current data on PPI usage in both urban and rural critical care environments and evaluate the current practices.

Rural vs urban dichotomy

There is limited medical research happening in rural America, making it more difficult for providers to effectively treat patients in these environments [10]. Treatment plans are extrapolated from data collected in urban research centers, despite an unmistakable difference in resource availability and patient demographics. Rural providers have fewer ICU beds at their disposal and a lack of specialist support, in addition to limited access to current research [10]. Residents of rural areas make up nearly 20% of the US population, but their local hospitals have only 1% of all ICU beds [11]. A general lack of accessible healthcare means that rural patients also have higher rates of comorbidities due to unmet needs and increased time and distance to treatment facilities [10-11]. Research from the COVID-19 pandemic showed that patients in rural hospitals have higher hospitalization and mortality rates as well as more days spent in the ICU [11]. The existing literature does not contain specific reports on PPI use in rural ICUs, nor does it make comparisons with urban ICUs, highlighting the need for additional research in this area.

## Review

Methods

**Figure 1:**
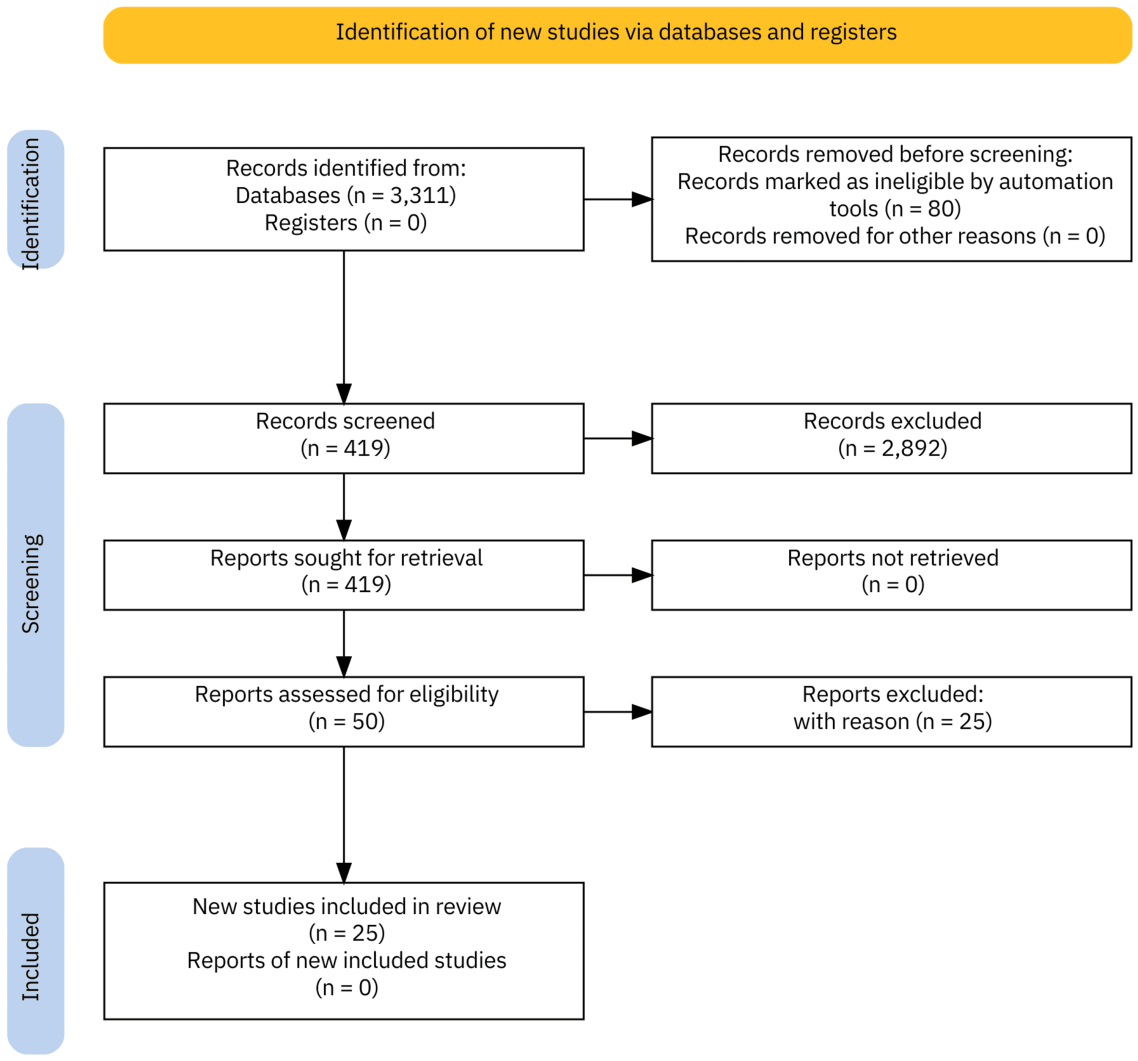
PRISMA flow diagram of the summarized search strategy. n: number of studies; PRISMA: Preferred Reporting Items for Systematic Reviews and Meta-Analyses

Data for this review were sourced from systematic reviews published on PubMed between May 1, 2024, and July 31, 2024. An advanced search was performed with the keyword “Proton Pump Inhibitors,” restricting results to systematic reviews published within the past 10 years to ensure relevance and recency. The initial search identified 505 systematic reviews. To refine the selection, the search was narrowed using the additional term “adverse effects,” which reduced the number of relevant reviews to 202. Eligibility criteria were then applied, leading to a final selection of 16 systematic reviews. Further, to complement the systematic reviews, stand-alone articles were sought using a series of specific search terms: “gastrointestinal bleeding AND critical care AND risk factor,” “hypotension AND incidence AND ICU,” “ICU bed availability AND rural,” and “rural hospitalization AND incidence, and “hypotension AND incidence”. This supplementary search identified eight additional articles. The 25 selected systematic reviews and stand-alone articles were thoroughly evaluated to assess the utilization of PPIs in critical care settings and to understand current standard practices. PubMed was the only electronic database used for this review. Our assessment encompassed various study types, including clinical trials, meta-analyses, systematic reviews, and observational studies, in order to gain a comprehensive understanding of the current data on PPI use (Table 1). Each study type presented unique strengths - clinical trials provided robust, controlled data; meta-analyses and systematic reviews synthesized a broad range of findings, enhancing generalizability; and observational studies offered real-world insights. This combination enabled a more nuanced interpretation of the evidence and reduced the potential for bias associated with reliance on a single research approach. Figure 1 presents a flow diagram of the search strategy utilized for this literature review.

**Table 1:** Summary of findings from included research studies RCS: retrospective cohort study; NR: narrative review; RP: recommendations proposal; BC: book chapter; POS: prospective observational study; PCS: prospective cohort study; SR: systematic review; MA: meta-analysis

Study	Population considered/number of studies reviewed	Study design	Main findings/outcome	Limitations/gaps in literature	
Orelio et al. [1]	14 studies	SR	Reduction in inappropriate use for SUP in hospitalized patients, with secondary outcomes including any potential adverse effects of de-implementation	Heterogeneity of studies, quality of included studies, lack of standardized outcome measures	
Grube and May [2]	N/A	NR	Use of SUP in non-ICU patients is not based on strong evidence emphasizing a need for better-defined criteria for SUP use in non-ICU settings.	Lack of high-quality evidence, potential overuse, inconsistent guidelines	
Farrell et al. [3]	N/A	RP	The guideline provides recommendations for deprescribing PPIs in patients who no longer require them.	Variability in study designs/populations, limited high-quality evidence, focus on short-term outcomes	
Shanika et al. [4]	89 contributing studies	SR	Global increase and inappropriate use of PPIs with variation in guideline adherence and with cost implications	Heterogeneity of data, quality of evidence, lack of standardized definitions	
Anzalone et al. [5]	General population (n = 1,033,229)	RCS	Higher hospitalization and mortality rates for rural residents during the COVID-19 pandemic	Rural residents are over 80% white	
Davoodi et al. [6]	4 peer-reviewed papers, 12 non-peer-reviewed sources, 6 US government sources	NR	Rural communities lack ICU beds and resources to care for older adult patients	Collected county-specific data and extrapolated to state-wide reports	
Ben-Eltriki et al. [7]	13 contributing studies	MA	Impact of PPI use on mortality may not be as substantial as sometimes suggested	Heterogeneity of included studies, quality of observational studies, short duration of follow-up	
Ribiere et al. [8]	N/A	NR	Clarifying long-term risks associated with PPI use such as kidney disease, bone fractures, GI fractions, and other adverse reactions	Variability in study quality, inconsistent definitions & outcomes, lack of standardization, translation from French	
Lambert et al. [9]	13 contributing studies	MA	Association of PPI use and an increased risk of community-acquired pneumonia (CAP)	Observational studies, heterogeneity, confounding factors	
Cheungpasitporn et al. [10]	9 contributing studies	MA	Association between PPI use and increased risk of hypomagnesemia	Study design limitations, heterogeneity, quality of included studies	
Swarnakari et al. [11]	15 contributing studies	SR	Long-term use of PPIs is associated with higher risk of vitamin B12 deficiency	Heterogeneity, quality of evidence, long-term effects	
Gao et al. [12]	N/A	MA	Association between PPI use and increased risk of gastric cancer	Heterogeneity of included studies, quality of primary studies, publication bias	
Jiang et al. [13]	13 contributing studies	MA	Association between long-term PPI use and increased risk of gastric cancer	Heterogeneity of included studies, quality of evidence, confounding factors	
Lv et al. [14]	N/A	MA	Potential association between prolonged PPI use and gastric precancerous conditions	Study quality and heterogeneity, limited long-term data, generalizability	
Strand et al. [15]	N/A	NR	PPI are effective for their indicated conditions, long-term use associated with potential risks of nutrient deficiencies, risk of infections, potential renal complications; increased PPI use over the years	Lack of high-quality evidence, inconsistency in guidelines, emerging risk	
Song et al. [16]	16 contributing studies	MA	Increased risk of mortality associated with PPI use among the elderly population	Observational studies, heterogeneity, potential confounders	
Nevalainen Nevalainen [17]	22 contributing studies	MA	Association between PPI use and increased risk of autoimmune and immune-mediated inflammatory diseases	Heterogeneity, potential bias, confounding factors	
Blackett et al. [18]	General population (n = 24,751)	RCS	Many patients are started on PPIs in the ICU without indication and continued after discharge	Patients with any indication of PPI use at the beginning of their stay were excluded	
Buendgens et al. [19]	10 contributing studies	RP	Use of PPIs must be balanced between risks and benefits	No differentiation between SMRD-related bleeding and CSGIB	
Shuman et al. [20]	16 contributing studies	NR	Both PPIs and H2-blockers are more effective than placebo at preventing CSGIB.	Only includes patients not on SUP.	
Cook et al. [21]	General population (n = 2252)	PCS	SUP is only required in patients needing mechanical ventilation or with coagulopathy	Enrollment was closed on weekends; physicians were only encouraged, not required to withhold SUP for study participants.	
Bořilová Linhartová et al. [22]	General population (specifically European, American, and African people)	RP	PPIs can be prescribed with a lower risk for adverse risks based on genetic profiling.	Recommendations mainly valid only for those of European, American, and African descent	
Mahajan and Prabhakar [23]	N/A	BC	Defines coagulopathy, its causes, and potential management	Presented in the context of neuroanesthesia	
Scherbring [24]	Adapted from “Stomach with Callout (Layout)”	Figure	Maps out the pathophysiology of stress-related mucosal disease from hemodynamic compromise to CSGIB	Retrieved from https://app.biorender.com/biorender-templates	
Terwindt et al. [25]	General population (n = 499)	POS	The vast majority of ICU patients experience systemic hypotension during their stay.	Any occurrence of hypotension longer than 10 seconds was included in the analysis.	
Lo et al. [26]	16 contributing studies	SR	Limited evidence supporting the use of PPIs in the management of gastroesophageal varices	Quality of evidence, inconsistent definitions and measures, limited data	

Literature review

To understand the usage of PPIs in both rural and urban critical care environments, it was necessary to conduct a thorough review of the literature on PPI indications, risks, and appropriate and inappropriate continuation. The literature emphasized the significance of the overutilization of PPIs and the associated risks for patient populations.

Risks of SUP

Prolonged and extensive use of PPIs has raised concerns regarding potential adverse effects and increased mortality rates, particularly in long-term use and specific patient populations with certain comorbidities. While PPIs were initially approved for short-term use within two to eight weeks, higher adverse events have been associated with their use [12]. These adverse events included increased risk of infections, gastric or intestinal cancer, digestive/malabsorption risk, chronic kidney disease, and other potential mechanisms such as dementia, osteoporosis, and cardiovascular risks [8, 12-19].

The research assessed older populations taking PPIs, which revealed a median exposure time of 1 to 4.6 years and a higher risk of serious mortality, primarily related to cardiovascular and chronic kidney disease deaths [12]. The findings were limited in their generalizability due to a predominantly male population, with 96% in randomized clinical trials (RCT) and 78% in cohort studies. Additionally, the sample was primarily Caucasian, with 87% in RCT and 60% in the cohort studies [20]. Similarly, another study found that older populations (aged 50 or older) showed an increased risk of death associated with PPI use in the cohort and long-term meta-analysis, even though there was no difference in the meta-analysis of RCTs [20]. PPI use was also linked to an elevated risk of infections such as community-acquired pneumonia (CAP), with a 1.5 times higher risk, particularly within the first 30 days of use (odds ratio (OR) 2.10, 95% CI 1.39 to 3.16) [14]. However, it is important to note that the studies included in these meta-analyses had overlapping populations of subjects and varying inclusion criteria for eligible participants [14].

Furthermore, PPI use increased the risk of mortality in patient populations with cirrhosis, and it has been repeatedly associated with hypomagnesemia, leading to the risk of related cardiovascular events (OR 1.43, 95% CI 1.08-1.88) [15]. These findings should be carefully considered given the selection bias and confounding variables in these observational studies. PPI use is also associated with vitamin deficiencies, particularly vitamin B12, which can lead to elevated homocysteine and methylmalonic acid levels, increasing the risk of macrocytic anemia and elevated cardiovascular events. The limitations of this study are attributed to the absence of RCTs and reliance on observational studies [16].

PPI use was also linked to the development of several cancers, including gastric cancer even after the eradication of *Helicobacter pylori*, as well as abnormal precancerous lesions such as intestinal metaplasia [17-19]. Further research is necessary as there are currently a limited number of meta-analyses, and determining causality between PPIs and cancer requires additional investigation.

Research has indicated to use PPIs cautiously in patient populations with autoimmune diseases, pancreatitis, rheumatoid arthritis, colitis, and other immune-mediated or inflammatory diseases [9]. However, due to the limited number of prospective cohort studies and small sample sizes, more effort is needed to improve understanding in future research.

In the last 30 years, PPIs have been proven to reduce bleeding events, but the number of patients who receive them far exceeds the number whose benefits outweigh the risks [1,3-4, 6-7, 21]. Researchers have repeatedly shown that a subset of patients with specific risk factors profit most from PPI usage in the ICU [6-7,21].

Risk Factors for GI Bleeding in the ICU

CSGIB events in the ICU resulting from stress ulcers have decreased as medicine evolves, especially noted over the past few decades [6-7]. Studies reported rates of CSGIB as high as 17% in 1987 and as low as 2 to 3% in 2021 [7,21]. Accuracy of the percentages is limited due to the study from 1987 only including patients not on SUP and recent approximations including ICU patients with and without SUP [6-7,21]. In addition, extrapolating results from these studies was complicated by varying definitions or no definition at all of CSGIB [2,6-7,22]. The definitions that do exist in the literature can be summarized as overt bleeding associated with hemodynamic compromise or requiring a blood transfusion [2,6-7,22].

While studies do not necessarily agree on the definition of CSGIB, there is consensus on the most important factors for increased risk of CSGIB in the ICU [2,6-7,22]. The most consistent high-risk factors were respiratory failure with mechanical ventilation and coagulopathy (including history of GI bleeding) [2,6-7,22]. The literature has consistently found these two high-risk factors to be the most significant factors for over two decades [2,22]. Mechanical ventilation may decrease splanchnic blood flow, allowing for ischemic damage and increasing the risk of bleeding [6]. Coagulopathy is an impairment of clotting ability that predisposes patients to bleeding in general [23]. Other notable risk factors include shock of any type, end-stage kidney disease on dialysis, and liver disease, especially if treated with PPIs [6-7,22]. A possible increased risk level was presented for patients with three or more comorbidities complicating the ICU stay [6]. Shock and kidney and liver disease may be secondary risk factors placing patients at increased risk for CSGIB by interfering with the normal physiology of the body, including blood pressure modulation via nitric oxide and endothelin-1 [6].

Pathophysiology of Stress-Related Mucosal Disease

The majority of patients in the ICU have some signs of stress-related mucosal disease (SMRD) due to local or systemic hemodynamic compromise [6,22]. SMRD is the inciting influence for stress ulcers and, therefore, CSGIB [6]. Systemic hypotension resulting from shock or other circumstances is a frequent admission diagnosis or complicating factor during ICU stays [25]. Mechanically ventilated patients may experience local changes in splanchnic blood flow because of the positive end-expiratory pressure (PEEP) used to maintain alveolar recruitment [6]. Reduction of gastric mucosal blood flow modifies the release of nitric oxide and endothelin-1 intravascularly. Nitric oxide, a potent vasodilator, decreases and endothelin-1, a vasoconstrictor, increases in an attempt to locally increase blood pressure [6]. Instead, this results in ischemia, harming the gastric mucosa [6]. Diminished splanchnic blood supply causes SMRD and can lead to ulceration and eventual CSGIB if not mitigated by pharmacologic or natural physiologic means [6].

In a healthy stomach, protective mechanisms act to maintain tolerable levels of gastric acid secretion and preserve the mucosa. In rat models, ischemia caused by gastric hypoperfusion led to inhibition of both derivative pathways of arachidonic acid [6]. Prostaglandins and leukotrienes, most importantly PGE2, were critical for gastric protection as they inhibited gastric acid production and stimulated gastric mucus and bicarbonate production [6]. The gastric mucosa was left open to damage due to the decreased blood flow, however, the reduction of gastric acid production as well as neutralization by bicarbonate acted as innate protection. Increased gastric mucus impeded deterioration of the tissue, delaying the onset of SMRD. This study did not, however, explore the effects of PPIs on the physiological responses [6].

**Figure 2:**
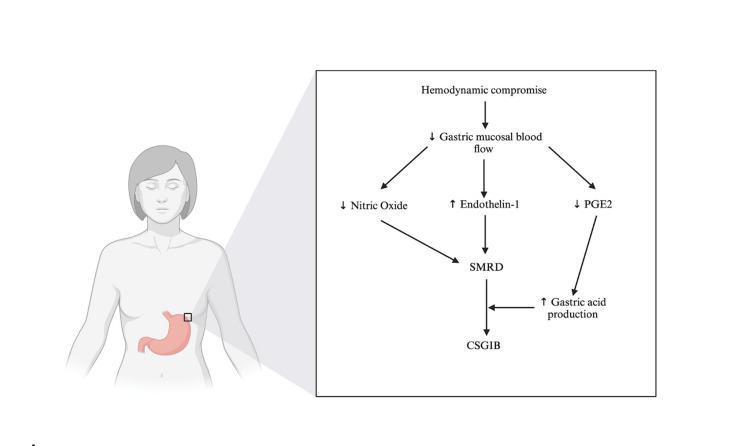
Pathophysiology of Stress-Related Mucosal Disease PGE2: prostaglandin E2; SMRD: stress-related mucosal disease; CSGIB: clinically significant gastrointestinal bleeding Adapted from “Stomach with Callout (Layout),” by Biorender.com (2024). Retrieved from https://app.biorender.com/biorender-templates [24].

PPIs act as a pharmacological adjunct to the physiologic response of PGE2. They suppress gastric acid production allowing other protective physiologic mechanisms to work more effectively [6]. PPIs do not directly affect the pathophysiology of SMRD but do have a key role in stress ulcer prevention in the setting of high-risk patients [6]. Their benefits may be eclipsed by adverse effects when inappropriately continued beyond the necessary therapeutic duration (Figure 2) [3,5].

Appropriate and Inappropriate PPI Continuation

It is crucial to establish protocols and standardize PPI usage, especially considering the increasing evidence of the numerous adverse effects associated with their use [1,3,5]. Current standard practices for PPI usage mainly focus on addressing acid-related and gastrointestinal tract disorders, such as reflux disease, peptic ulcer disease, and SUP for high-risk patients. PPIs are first-line agents for treating esophagitis, non-erosive reflux disease, peptic ulcer disease, preventing NSAID-induced ulcers, managing Zollinger-Ellison Syndrome, and as part of therapy for *H. pylori* infections [8]. However, there is a growing global trend of off-label and inappropriate use [5]. The inappropriate use often relates to the length of treatment, the age of patients, and the presence of various other health conditions in certain patients [3,12].

The long-term use of PPIs is a significant concern, as it has been associated with higher mortality rates and poor health outcomes due to serious adverse events (SAEs), particularly in older adults aged 50 years or older, who face a 15% higher risk of mortality compared to nonusers (Risk ratio (RR) 1.15, 95% CI 1.10-1.20 [20]. According to current research, the best outcomes from PPI use were observed with short-term courses (e.g., 10 days), and higher dose infusions and prolonged use were not recommended (OR 2.77, 95% CI 1.82-4.23) [26]. Limitations regarding these recommendations include limited high-quality studies, mostly relying on small or retrospective trials.

Age also served as a critical factor in determining PPI use. The risk of mortality associated with PPIs increases after the age of 50, possibly due to higher comorbidities and worsening health status, which drastically elevates the risk of adverse effects from these medications [12]. There is limited generalizability of these findings due to the predominance of only Caucasian male subjects. Furthermore, individuals with other health conditions or comorbidities such as organ failure (cirrhosis, chronic kidney disease, cardiovascular disease), autoimmune diseases, and inflammatory diseases should exercise caution when using PPIs, opting for shorter courses, lower doses, or alternative treatments [3,9,26].

Considering these findings, the standardization of PPI use must be improved to reduce harm and treatment costs. Recent research examined global trends and practices of PPI use, which revealed that unnecessarily prolonged use was a major issue leading to poorer health outcomes [3,5]. This literature recommended healthcare providers regularly review PPI prescriptions and discontinue them when they are no longer deemed appropriate or beneficial [3,5].

Another study aimed to find ways to reduce the inappropriate use of PPIs for SUP in hospitalized patients. The authors conducted a systematic review of de-implementation studies focusing on reducing or stopping low-value healthcare practices. The strategies included educational programs, guideline implementation, audit and feedback, and electronic health record (EHR) modifications. It was found that multifaceted approaches combining all these intervention types tend to be the most successful. However, the most effective intervention was education targeted at healthcare providers. A major limitation of the study was the terminology used to describe de-implementation strategies, which varies widely and may lead to relevant studies being missed [1].

Future research

The current literature available for PPI usage in the ICU is comprehensive for an urban ICU setting and offers standardization procedures. There is a distinct lack of research assessing the differences between urban and rural ICU usage and the effects of resource availability. A study comparing PPI usage in rural and urban ICUs and offering clinical recommendations for identifying high-risk patients that acknowledge the difficulties faced in rural critical care medicine is proposed.

The existing literature on PPI usage in ICU settings is extensive and well-developed for urban environments, providing detailed standardization procedures. However, there is a notable gap in research exploring the differences in PPI usage between urban and rural ICUs, as well as the impact of resource availability on such practices. To address this gap, future research should investigate and compare PPI usage in rural vs urban ICUs. Future studies should aim to provide clinical recommendations for identifying high-risk patients while considering the unique challenges faced in rural critical care settings.

## Conclusions

PPIs have a longstanding role in the ICU as SUPs. Recent research has shown that instances of CSGIB have decreased over the last three decades with no clear correlation with SUP. However, most of the research originated from large urban academic centers or international sources and recommendations extrapolated for rural practice.

PPIs have not been proven to improve mortality rates for ICU patients with or without GIB. While PPIs do have clear indications in a subset of ICU patients, they are often overutilized leading to increased risk for long-term side effects. PPIs are one of the most commonly prescribed drugs in the world and are taken over-the-counter as well, but the risks outweigh the benefits for many patients. There is a lack of distinction between critically ill patients at high enough risk for CSGIB to benefit from PPI administration and those who are simply in the ICU. This review has also shown a need for standardization of PPI usage in the ICU setting that recognizes the contrast between rural and urban critical care.
